# Genetic characterization of two G8P[8] rotavirus strains isolated in Guangzhou, China, in 2020/21: evidence of genome reassortment

**DOI:** 10.1186/s12879-022-07542-9

**Published:** 2022-06-28

**Authors:** Si-Jie Wang, Li-Na Chen, Song-Mei Wang, Hong-Lu Zhou, Chao Qiu, Baoming Jiang, Tian-Yi Qiu, Sheng-Li Chen, Lorenz von Seidlein, Xuan-Yi Wang

**Affiliations:** 1 Shanghai Institute of Infectious Disease and Biosecurity, and Institutes of Biomedical Sciences, Fudan University, Shanghai, People’s Republic of China; 2 Key Laboratory of Medical Molecular Virology of MoE & MoH, Fudan University, Shanghai, People’s Republic of China; 3 Laboratory of Molecular Biology, Training Center of Medical Experiments, School of Basic Medical Sciences, Fudan University, Shanghai, People’s Republic of China; 4 Viral Gastroenteritis Branch, Division of Viral Diseases, National Center for Immunization and Respiratory Diseases, Centers for Disease Control and Prevention, Atlanta, GA USA; 5 Zhongshan Hospital, Shanghai Public Health Clinical Center, Fudan University, Shanghai, People’s Republic of China; 6 Pediatric Center, Zhujiang Hospital, Southern Medical University, 253 Industrial Avenue Central, Guangzhou, People’s Republic of China; 7 Mahidol-Oxford Tropical Medicine Research Unit, Faculty of Tropical Medicine, Mahidol University, Bangkok, Thailand; 8 Children’s Hospital, Fudan University, Shanghai, People’s Republic of China

**Keywords:** Gastroenteritis, Rotavirus, Emergence, China, Genotyping

## Abstract

**Background:**

The G8 rotavirus genotype has been detected frequently in children in many countries and even became the predominant strain in sub-Saharan African countries, while there are currently no reports from China. In this study we described the genetic characteristics and evolutionary relationship between rotavirus strains from Guangzhou in China and the epidemic rotavirus strains derived from GenBank, 2020–2021.

**Methods:**

Virus isolation and subsequent next-generation sequencing were performed for confirmed G8P[8] specimens. The genetic characteristics and evolutionary relationship were analyzed in comparison with epidemic rotavirus sequences obtained from GenBank.

**Results:**

The two Guangzhou G8 strains were DS-1-like with the closest genetic distance to strains circulating in Southeast Asia. The VP7 genes of the two strains were derived from a human, not an animal G8 rotavirus. Large genetic distances in several genes suggested that the Guangzhou strains may not have been transmitted directly from Southeast Asian countries, but have emerged following reassortment events.

**Conclusions:**

We report the whole genome sequence information of G8P[8] rotaviruses recently detected in China; their clinical and epidemiological significance remains to be explored further.

**Supplementary Information:**

The online version contains supplementary material available at 10.1186/s12879-022-07542-9.

## Background

Despite significant advances in fighting childhood diarrhea through sanitation improvement, and vaccine introduction, diarrheal diseases remained worldwide the fourth most frequent cause of death for children < 5 years of age in 2016 [1]. Rotavirus (RV) infection remains the leading cause of severe acute gastroenteritis (AGE) [2]. In countries where rotavirus mass vaccination programs have been established, noroviruses have taken over as the most frequent cause of childhood AGE [3–5]. The effectiveness of these vaccines and the decline in diarrheal disease caused by rotavirus has been faster in the developed countries but remains disappointingly low in the low-middle-income countries [1, 6–8]. Such differences in disease distribution and severity are likely driven by circulating rotavirus strains and genotype distributions [6, 8].

Rotaviruses are members of the genus *Rotavirus* within the family *Reoviridae*, with a genome of 11 segments of double-stranded RNA (dsRNA), encoding six structural viral proteins (VP1-4, VP6, and VP7) and six non-structural proteins (NSP1-5/6) [9]. Based on the sequences of VP4 and VP7 proteins, RVs are divided into different P (VP4) and G (VP7) genotypes, respectively. Globally, six G types (G1-4, G9, and G12) and three P types (P[4], P[6], and P[8]) have been predominant in the past decades[2, 10]. Data from the Chinese RV sentinel surveillance network showed that G1, G2, and G4 were very rarely reported since 2012, and the predominant G type in China was G9 (~ 90%), followed by G3 (~ 7%) [11]. The G8 genotype has not been reported before. In the present study, the whole genome sequences of G8 rotavirus strains in China are reported, and the genetic characteristics and evolutionary relationships between rotavirus strains from Guangzhou in China and epidemic rotavirus strains derived from GenBank are described.

## Materials and methods

### Participants and specimens Collection

A hospital-based study on children of less than 5 years of age, hospitalized for acute gastroenteritis caused by rotavirus infection, was conducted at Zhujiang Hospital, Guangzhou between December 2020 and February 2021. Stool specimens were collected from each patient with severe AGE at the admission for laboratory diagnosis as a routine clinical procedure. 16 fecal specimens were collected.

### Rotavirus antigen detection, RNA extraction and RT-PCR

A commercial enzyme immunoassay (EIA) (Ridascreen, R-Biopharm AG, Germany) was applied for RV detection [12]. Those RV antigen positive samples were further suspended in 1×phosphate-buffered saline (PBS; Invitrogen) to approximately 10%, and centrifuged for 10 minutes at 8000×g. Viral RNA was automatically extracted from 200 µL supernatant stool samples using the Nucleic Acid Extraction Kit (Tianlong, Xian, China) to be used in RT-PCR. Amplification of the partial VP7/VP4 genes were performed in a 25 µL reaction volume containing 10 µM forward primers Beg9 (5’- GGCTTTAAAAGAGAGAATTTCCGTCTGG-3’) or Con3 (5’-TGGCTTCGCTCATTTATAGACA-3’) and reverse primers End9 (5’-GGTCACATCATACAATTCTAATCTAAG − 3’) or Con2 (5’-ATTTCGGACCATTTATAACC-3’), respectively [13], and 2 µL RNA template using the FastKing One Step RT-PCR Kit (TIANGEN, Beijing, China). Reverse transcription was conducted at 42˚C for 30 min, followed by initial denaturation at 95˚C for 3 min. In total, 40 cycles of amplification were performed comprising denaturation at 94˚C for 30 s, annealing at 55˚C (VP7)/49˚C (VP4) for 30 s, and extension at 72˚C for 30 s; the program ended at 72˚C for 5 min. The PCR products were analyzed by electrophoresis in 1% agarose gels. Sanger sequencing of obtained amplicons (1062 bp for VP7 and 876 bp for VP4) was performed and analyzed with the BLAST database to determine the G and P genotypes.

### Whole-genome sequencing

Library preparation and Illumina sequencing were performed by a commercial provider (Tsingke, China). First, the nucleic acid was fragmented, and the average fragment size used for sequencing was 300∼500 bp. Paired-end (PE) 100-base sequencing was performed using Illumina Novaseq 6000 PE150. Fastp (version 0.20.0) and bbmap (version 38.51) were used as tools to remove adapter sequences and contaminating sequences in reads [14, 15]. The remaining reads were then subjected to de novo contig assembly using SPAdes (version 3.14.1) and SOAPdenovo (version 2.04), which assembles reads based on the de Bruijin graph algorithm [16, 17]. The generated contigs were then analyzed by BLAST (version 2.10.0+) using the NCBI nonredundant nucleotide (NT) and viral refseq databases to evaluate the accuracy and completeness of the obtained assembly results [18].

### Phylogenetic analysis of rotavirus G and P genotypes

Original data of strains and reference sequences that were more similar to the sequenced strains were downloaded from the GenBank database. DNAMAN (version 9.0) software was applied for sequence similarity analysis. Multiple alignments and phylogenetic analysis were performed with MEGA X software. The phylogenetic trees were constructed using the neighbor-joining method. Kimura-2 parameter model and gamma distribution were used to calculate genetic distance, and reliability analyses were performed using the bootstrap method, repeated sampling 1000 times, with less than 70% was considered meaningless. The percentages of nucleotide-sequence similarity between Guangzhou RVA strains and RVA sequences deposited in the GenBank were calculated using the p-distances method. [19]

### Construction of background RV strains

To compare the genome constellations of the G8P[8] isolates from Guangzhou with those of RVA mainly from Asia and Africa, complete genome sequences of 44 representative RVA strains available from GenBank were selected for comparison (Table 1). In addition, complete genome sequences of 47 representative RVA strains around the world which were available from GenBank were obtained in order to determine whether the Guangzhou strains had arisen following reassortment events.

**Table 1:** Comparison of genome constellations of the G8P[8] isolates from Guangzhou with those of RVA genomes available from GenBank

Strain	Origin	VP7	VP4	VP6	VP1	VP2	VP3	NSP1	NSP2	NSP3	NSP4	NSP5/6	Shared genotypes
RVA/Human-wt/CHN/GZ-0005/2021/G8P[8]	Human	G8	P[8]	I2	R2	C2	M2	A2	N2	T2	E2	H2	
RVA/Human-wt/CHN/GZ-0013/2021/G8P[8]	Human	G8	P[8]	I2	R2	C2	M2	A2	N2	T2	E2	H2	
RVA/Human-wt/THA/SSKT-269/2014/G8P[8]	Human	G8	P[8]	I2	R2	C2	M2	A2	N2	T2	E2	H2	11
RVA/Human-wt/THA/SSL-55/2014/G8P[8]	Human	G8	P[8]	I2	R2	C2	M2	A2	N2	T2	E2	H2	11
RVA/Human-wt/THA/SKT-457/2014/G8P[8]	Human	G8	P[8]	I2	R2	C2	M2	A2	N2	T2	E2	H2	11
RVA/Human-wt/THA/PCB-85/2013/G8P[8]	Human	G8	P[8]	I2	R2	C2	M2	A2	N2	T2	E2	H2	11
RVA/Human-wt/SGP/NV-16-124/2016/G8P[8]	Human	G8	P[8]	I2	R2	C2	M2	A2	N2	T2	E2	H2	11
RVA/Human-wt/VNM/RVN1326/2014/G8P[8]	Human	G8	P[8]	I2	R2	C2	M2	A2	N2	T2	E2	H2	11
RVA/Human-wt/JPN/17,287/2019/G8P[8]	Human	G8	P[8]	I2	R2	C2	M2	A2	N2	T2	E2	H2	11
RVA/Human-wt/JPN/SO1162/2017/G8P[8]	Human	G8	P[8]	I2	R2	C2	M2	A2	N2	T2	E2	H2	11
RVA/Human-wt/KOR/CAU17L-79/2017/G8P[8]	Human	G8	P[8]	I2	R2	C2	M2	A2	N2	T2	E2	H2	11
RVA/Human-wt/COD/DRC88/2003/G8P[8]	Human	G8	P[8]	I2	R2	C2	M2	A2	N2	T2	E2	H2	11
RVA/Human-wt/THA/DBM2017-203/2017/G9P[8]	Human	G9	P[8]	I2	R2	C2	M2	A2	N2	T2	E2	H2	10
RVA/Human-wt/THA/DBM2018-291/2018/G9P[8]	Human	G9	P[8]	I2	R2	C2	M2	A2	N2	T2	E2	H2	10
RVA/Human-wt/ESP/SS61921417/2015/G3P[8]	Human	G3	P[8]	I2	R2	C2	M2	A2	N2	T2	E2	H2	10
RVA/Human-wt/ESP/SS96217158/2015/G3P[8]	Human	G3	P[8]	I2	R2	C2	M2	A2	N2	T2	E2	H2	10
RVA/Human-wt/ESP/SS61720845/2015/G3P[8]	Human	G3	P[8]	I2	R2	C2	M2	A2	N2	T2	E2	H2	10
RVA/Human-wt/TWN/2014/103-701-D022/G3P[8]	Human	G3	P[8]	I2	R2	C2	M2	A2	N2	T2	E2	H2	10
RVA/Human-tc/IND/69 M/1980/G8P[10]	Human	G8	P[10]	I2	R2	C2	M2	A2	N2	T2	E2	H2	10
RVA/Human-wt/COD/DRC86/2003/G8P[6]	Human	G8	P[6]	I2	R2	C2	M2	A2	N2	T2	E2	H2	10
RVA/Human-wt/MLI/Mali-119/2008/G8P[6]	Human	G8	P[6]	I2	R2	C2	M2	A2	N2	T2	E2	H2	10
RVA/Human-wt/UGA/MUL-13-308/2013/G8P[6]	Human	G8	P[6]	I2	R2	C2	M2	A2	N2	T2	E2	H2	10
RVA/Human-wt/MWI/BID1B9/2012/G8P[4]	Human	G8	P[4]	I2	R2	C2	M2	A2	N2	T2	E2	H2	10
RVA/Human-wt/KEN/KDH1111/2011/G8P[4]	Human	G8	P[4]	I2	R2	C2	M2	A2	N2	T2	E2	H2	10
RVA/Human-wt/USA/2,009,727,045/2009/G8P[4]	Human	G8	P[4]	I2	R2	C2	M2	A2	N2	T2	E2	H2	10
RVA/Human-wt/THA/DBM2018-105/2018/G2P[4]	Human	G2	P[4]	I2	R2	C2	M2	A2	N2	T2	E2	H2	9
RVA/Human-tc/USA/DS-1/1976/G2P[4]	Human	G2	P[4]	I2	R2	C2	M2	A2	N2	T2	E2	H2	9
RVA/Human-wt/GHA/GH019-08/2008/G8P[6]	Human	G8	P[6]	I2	R2	C2	M2	A2	N2	T2	E2	H3	9
RVA/Human-wt/PAK419/2016/G3P[4]	Human	G3	P[8]	I2	R2	C2	M2	A2	N2	T1	E2	H2	9
RVA/Human-wt/USA/2,014,737,554/2014/G1P[8]	Human	G1	P[8]	I2	R2	C2	M2	A2	N2	T2	E2	H1	9
RVA/Cow-wt/ZAF/1604/2007/G8P[1]	Bovine	G8	P[1]	I2	R2	C2	M2	A3	N2	T6	E2	H3	7
RVA/Cow-tc/THA/A5-13/1988/G8P[1]	Bovine	G8	P[1]	I2	R2	C2	M2	A14	N2	T6	E2	H3	7
RVA/Human-wt/US/2,012,841,174/2012/G8P[14]	Human	G8	P[14]	I2	R3	C2	M2	A3	N2	T6	E2	H3	6
RVA/Human-wt/HRV/CR2006/2006/G8P[8]	Human	G8	P[8]	I1	R1	C1	M1	A1	N1	T1	E1	H1	2
RVA/Human-wt/TUN/6862/2000/G8P[8]	Human	G8	P[8]	I1	R1	C1	M1	A1	N1	T1	E1	H1	2
RVA/Human-wt/CHN/km15119/2016/G9P[8]	Human	G9	P[8]	I1	R1	C1	M1	A1	N1	T1	E1	H1	1
RVA/Human-wt/MLI/Mali-138/2008/G9P[8]	Human	G9	P[8]	I1	R1	C1	M1	A1	N1	T1	E1	H1	1
RVA/Human-lab/USA/Wa/2020/G1P[8]	Human	G1	P[8]	I1	R1	C1	M1	A1	N1	T1	E1	H1	1
RVA/Human-wt/ZAF/UFS-NGS-MRC-DPRU4269/2002/G1P[8]	Human	G1	P[8]	I1	R1	C1	M1	A1	N1	T1	E1	H1	1
RVA/Human-wt/CHN/E5365/2017/G1P[8]	Human	G1	P[8]	I1	R1	C1	M1	A1	N1	T1	E1	H1	1
RVA/Human-wt/CHN/E5867/2018/G3P[8]	Human	G3	P[8]	I1	R1	C1	M1	A1	N1	T1	E1	H1	1
RVA/Human-wt/UGA/NSA-13-043/2013/G9P[8]	Human	G9	P[8]	I1	R1	C1	M1	A1	N1	T1	E1	H1	1
RVA/Human-wt/USA/3,000,357,125/2016/G9P[8]	Human	G9	P[8]	I1	R1	C1	M1	A1	N1	T1	E1	H1	1
RVA/Human-wt/USA/3,000,354,346/2015/G12P[8]	Human	G12	P[8]	I1	R1	C1	M1	A1	N1	T1	E1	H1	1
RVA/Human/CHN/WZ606/2013/G3P[9]	Human	G3	P[9]	I3	R3	C3	M3	A3	N3	T3	E3	H3	0
RVA/Human- tc/JPN/AU-l/1982/G3P[9]	Human	G3	P[9]	I3	R3	C3	M3	A3	N3	T3	E3	H3	0

## Results

### Genotypes and genetic characteristics of Guangzhou G8 rotavirus

Between December 2020 and February 2021, 16 children < 5 years were hospitalized for treatment of severe AGE in Zhujiang Hospital, Guangzhou. Five of sixteen samples collected from hospitalized AGE children were positive for rotavirus. Further G/P typing differentiated the samples as two G8P[8] strains, two G9P[8] strains and one G2P[4] strain.

### Whole-genome sequencing

GenBank files containing genome sequences can be retrieved from GenBank (accession no. OK349178 - OK349199) (Additional file 1: Table S1). The whole-genome analysis confirmed that the two G8P[8] strains were DS-1-like strains with a genotype constellation of G8-P[8]-I2-R2-C2-M2-A2-N2-T2-E2-H2 (Table 1). The whole genome of the two G8 strains were highly similar with an overall genome identity of 99.78% and the sequence identity of 11 genome segments ranged from 99.47 to 99.96%.

### Large evolutionary distance between two Guangzhou G8P[8] strains and other circulating G8P[8] strains

Ten representative G8P[8] strains isolated in other areas shared the same genotype in all 11 genome segments with the two Guangzhou strains (Table 1). Of these, eight G8P[8] strains isolated between 2013 and 2019 were analyzed. The whole genome sequence identity between the eight G8P[8] strains and the two studied strains varied from 87.23 to 95.21%. For the segments encoding VP2, VP3, VP4, VP7, NSP1, and NSP3 sequence identities of > 98% were observed whereas the sequence identities of the other segments were lower (Table 2).

**Table 2:** Nucleotide sequence similarity of strains closely related to Guangzhou strains

Strain (genotype representative from study/country)	Genotype constellations and nucleotide identities (%), by gene^a, c^											
	**VP1**	**VP2**	**VP3**	**VP4**	**VP6**	**VP7**	**NSP1**	**NSP2**	**NSP3**	**NSP4**	**NSP5/6**	**Overall similarity**
Human/THA/SSKT-269/2014/G8P[8]	86.07	**98.80**	**98.30**	**98.05**	96.83	**99.80**	**98.85**	86.69	**98.41**	91.01	91.91	94.97
	86.00	**98.84**	**98.38**	**98.13**	96.83	**99.61**	**98.53**	86.78	**98.41**	90.57	91.79	94.92
Human/THA/SSL-55/2014/G8P[8]	85.94	**98.58**	**98.49**	**98.05**	96.83	**99.06**	**98.79**	86.62	**98.31**	91.22	97.06	95.21
	85.88	**98.62**	**98.57**	**98.09**	96.83	**98.87**	**98.47**	86.71	**98.31**	90.69	96.94	95.17
Human/THA/SKT-457/2014/G8P[8]	85.90	**98.80**	**98.11**	**98.13**	94.10	**98.78**	**98.08**	86.59	**98.03**	90.15	90.44	94.47
	85.90	**98.80**	**98.11**	**98.13**	94.10	**98.78**	**98.08**	86.59	**98.03**	90.15	90.44	94.47
Human/THA/PCB-85/2013/G8P[8]	85.93	**98.95**	**98.65**	**98.22**	91.30	**99.06**	**98.98**	86.69	**98.41**	93.87	97.55	94.96
	85.87	**98.99**	**98.73**	**98.30**	91.15	**98.87**	**98.66**	86.78	**98.41**	93.34	97.43	94.91
Human/JPN/17287/2019/G8P[8]	86.04	**98.62**	**98.07**	**98.01**	96.83	**98.78**	**98.79**	86.12	**98.03**	90.55	94.85	94.90
	85.97	**98.65**	**98.15**	**98.09**	96.68	**98.59**	**98.47**	86.21	**98.22**	90.01	94.73	94.85
Human/JPN/SO1162/2017/G8P[8]	93.32	**98.20**	96.91	97.67	95.65	92.84	97.89	85.58	95.59	75.53	94.36	94.65
	93.16	**98.24**	96.99	97.67	95.50	92.66	97.57	85.67	95.78	75.40	94.24	94.60
Human/KOR/CAU17L-79/2017/G8P[8]	85.67	97.80	96.60	97.97	92.70	**99.15**	95.60	82.47	95.59	90.43	94.73	93.72
	85.61	97.84	96.68	**98.05**	92.55	**98.96**	95.53	82.56	95.78	89.89	94.61	93.70
Human/SGP/NV-16-124/2016/G8P[8]^b^	92.82	79.58	93.05	96.19	89.97	90.68	93.94	82.75	62.20	84.57	59.19	87.30
	92.67	79.61	93.13	96.27	89.82	90.49	93.62	82.75	62.29	84.04	59.07	87.23
Human/THA/DBM2018-105/2018/G2P[4]	**99.57**	–	–	–	–	–	–	–	–	–	–	–
	**99.48**	–	–	–	–	–	–	–	–	–	–	–
Human/THA/DBM2017-203/2017/G9P[8]	–	**99.51**	–	–	–	–	–	–	–	–	–	–
	–	**99.55**	–	–	–	–	–	–	–	–	–	–
Human/ESP/SS61921417/2015/G3P[8]	–	–	**99.50**	–	–	–	–	–	–	–	–	–
	–	–	**99.50**	–	–	–	–	–	–	–	–	–
Human/THA/DBM2018-291/2018/G9P[8]	–	–	–	**99.45**	–	–	–	–	–	–	**99.75**	–
	–	–	–	**99.53**	–	–	–	–	–	–	**99.63**	–
Human/ESP/SS96217158/2015/G3P[8]	–	–	–	–	–	–	**99.62**	–	–	–	–	–
	–	–	–	–	–	–	**99.30**	–	–	–	–	–
Human/THA/DBM2017-016/2017/G9P[8]	–	–	–	–	**99.48**	–	–	–	–	–	–	–
	–	–	–	–	**99.48**	–	–	–	–	–	–	–
Human/ESP/SS61720845/2015/G3P[8]	–	–	–	–	–	–	–	**99.43**	**99.06**	**99.45**	–	–
	–	–	–	–	–	–	–	**99.53**	**99.06**	**98.91**	–	–
Human/THA/SSKT-269/2014/G8P[8]	–	–	–	–	–	**99.34**	–	–	–	–	–	–
	–	–	–	–	–	**99.15**	–	–	–	–	–	–

GenBank accession numbers used in this comparison were as follows: for SSKT-269, LC169951 (VP1), LC169952 (VP2), LC169953 (VP3), LC169954 (VP4), LC169955. (VP6), LC169956 (VP7), LC169957 (NSP1), LC169958 (NSP2), LC169959 (NSP3), LC169960 (NSP4), and LC169961 (NSP5/6); for SSL-55, LC169962 (VP1), LC169963 (VP2), LC169964 (VP3), LC169965 (VP4), LC169966 (VP6), LC169967 (VP7), LC169968 (NSP1), LC169969 (NSP2), LC169970 (NSP3), LC169971 (NSP4), and LC169972 (NSP5/6); for SKT-457, LC169940 (VP1), LC169941 (VP2), LC169942 (VP3), LC169943 (VP4), LC169944 (VP6), LC169945 (VP7), LC169946 (NSP1), LC169947 (NSP2), LC169948 (NSP3), LC169949 (NSP4), and LC169950 (NSP5/6); for PCB-85, LC169874 (VP1), LC169875 (VP2), LC169876 (VP3), LC169877 (VP4), LC169878 (VP6), LC169879 (VP7), LC169880 (NSP1), LC169881 (NSP2), LC169882 (NSP3), LC169883 (NSP4), and LC169884 (NSP5/6); for 17287, MT410495 (VP1), MT410496 (VP2), MT410497 (VP3), MT410498 (VP4), MT410499 (VP6), MT410500 (VP7), MT410490 (NSP1), MT410491 (NSP2), MT410492 (NSP3), MT410493 (NSP4), and MT410494 (NSP5/6); for SO1162, LC386065 (VP1), LC386066 (VP2), LC386067 (VP3), LC386068 (VP4), LC386069 (VP6), LC386070 (VP7), LC386071 (NSP1), LC386072 (NSP2), LC386073 (NSP3), LC386074 (NSP4), and LC386075 (NSP5/6); for CAU17L-79, MN058735 (VP1), MN058736 (VP2), MN058737 (VP3), MN058738 (VP4), MN058739 (VP6), MN058740 (VP7), MN058730 (NSP1), MN058731 (NSP2), MN058732 (NSP3), MN058733 (NSP4), and MN058734 (NSP5/6); for NV-16-124, G996057 (VP1), MG996065 (VP2), MG996073 (VP3), MG996081 (VP4), MG996089 (VP6), MG996097 (VP7), MG996105 (NSP1), MG996113 (NSP2), MG996121 (NSP3), MG996129 (NSP4), and MG996137 (NSP5/6); for DBM2018-105, LC514525 (VP1); for DBM2017-203, LC514482 (VP2); for SS61921417, KU550277 (VP3); for DBM2018-291, LC514495 (VP4), LC514502 (NSP5/6); for SS96217158, KU550302 (NSP1); for DBM2017-016, LC514474 (VP6); for SS61720845, KU550305 (NSP2), KU550311 (NSP3), KU550317 (NSP4); for SSKT-269, LC169956 (VP7)

^a^Bold indicates nucleotide identities >98.0%

^b^Percent identity based on partial available gene sequences

^c^Numbers indicate percent of nt identity between the genes of strains GZ-0005 (upper number) and GZ-0013 (lower number) and cogent genes of closest strains

### Phylogenetic analysis of G8P[8] genotype VP7, VP4 segments

**Fig. 1:**
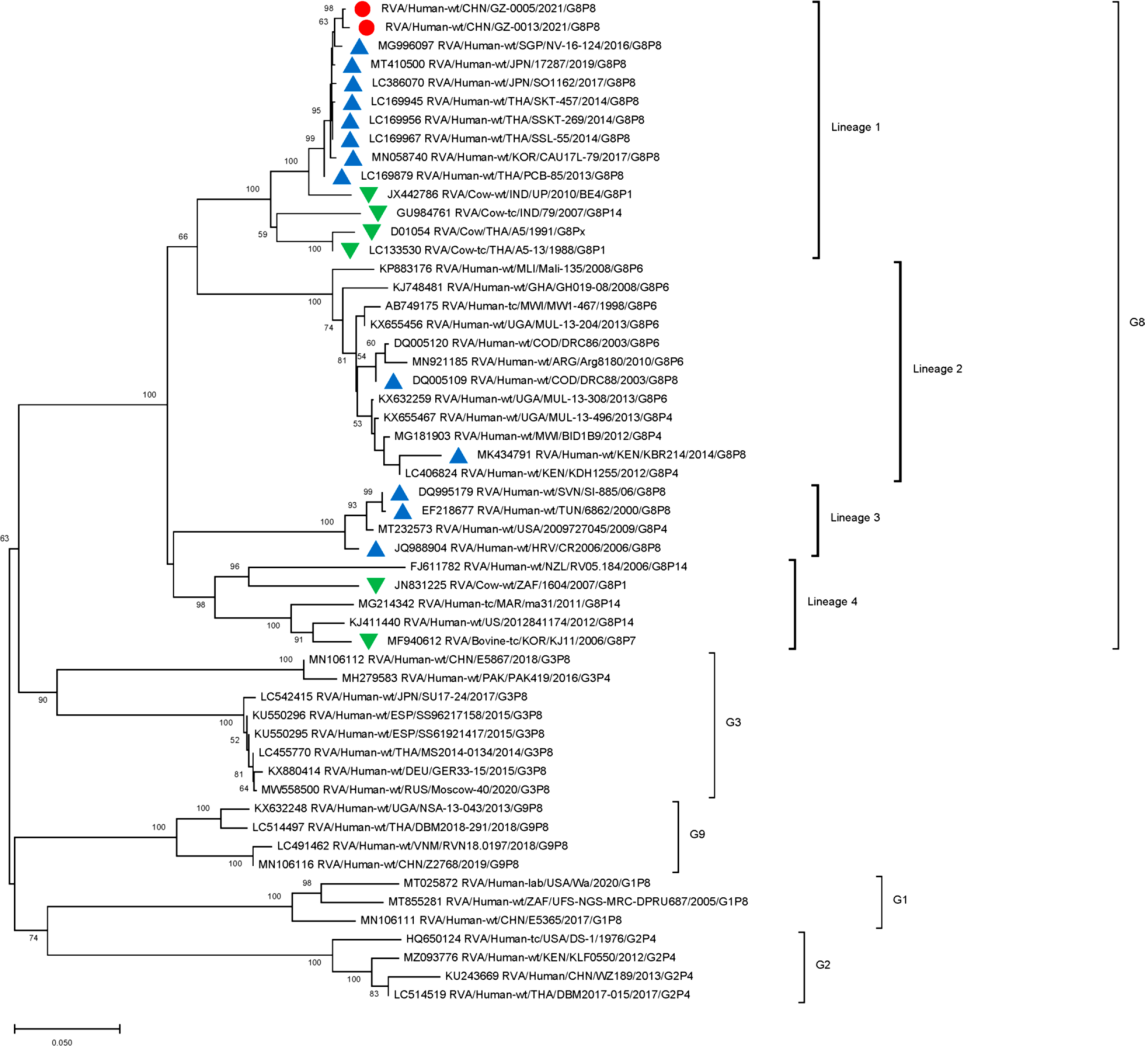
Phylogenetic analysis of the VP7 gene of G8 rotavirus strains used in the phylogenetic study of RVA strains. Phylogenetic tree of VP7 gene. This tree involves five different VP7 genes including G8, G3, G9, G1 and G2. The G8 gene was further clustered into four lineages. Studied strains were marked in different shapes and colors.  : sample strains;  : G8P[8] strains in various regions;  : animal-derived strains;  : strains in mainland China. All sequences except those of the sample strains were obtained from the NCBI public database. The Kimura-2 parameter model was used for the construction of the Neighbor-Joining phylogenetic tree. Bootstrap numbers are shown at the branch nodes and are more reliable at values of > 70%. The scale bar indicates nucleotide substitutions per site

We used the full length of the VP7 and VP4 gene sequences to construct phylogenetic trees. The nucleotide similarities of the two Guangzhou G8 strains and the Thailand strain (SSKT-269/THA/G8P[8]) were 99.34% and 99.15% (Table 2). In the phylogenetic tree of VP7 genes, the two Guangzhou strains were clustered exclusively with DS-1-like G8 strains formerly isolated and described in multiple regions such as Singapore, Japan, Thailand, and Korea (lineage 1). In addition, several G8 bovine rotaviruses in Southeast Asia (BE4/IND/G8P[1], 79/IND/G8P[14], A5-13/THA/G8P[14], A5/THA/G8Px) were also located on lineage (1) Other clusters of G8 genotypes in lineage 3 were obtained with Wa-like RVA strains, which were isolated in America (2,009,727,045/USA/G8P[4]), African countries (6862/TUN/G8P[8]), and European countries (CR2006/HRV/G8P[8], SI-885/SVN/G8P[8]). Other African DS-1-like G8 strains were clustered into a distinct lineage (2) (Fig. 1)

Further analysis indicated that the VP7 nucleotide sequence similarities between the Guangzhou G8 strains and the DS-1-like G8 strains in Southeast Asia were very close (differences 0.0066 ~ 0.0114). However, the genetic distances between the Guangzhou strains and the DS-1-like G8 strains in Africa were further apart (differences 0.1476 ~ 0.1678). In addition, the genetic distances between the two Guangzhou strains and the Wa-like G8 strains were even further apart with a genetic distance over 0.1689 (Additional file 1: Table S2). The sequence identities between the Guangzhou strains and bovine strains ranged from 97.51 to 98.66%, lower than those between the Guangzhou strains and other human G8 strains (over 99%).

**Fig. 2:**
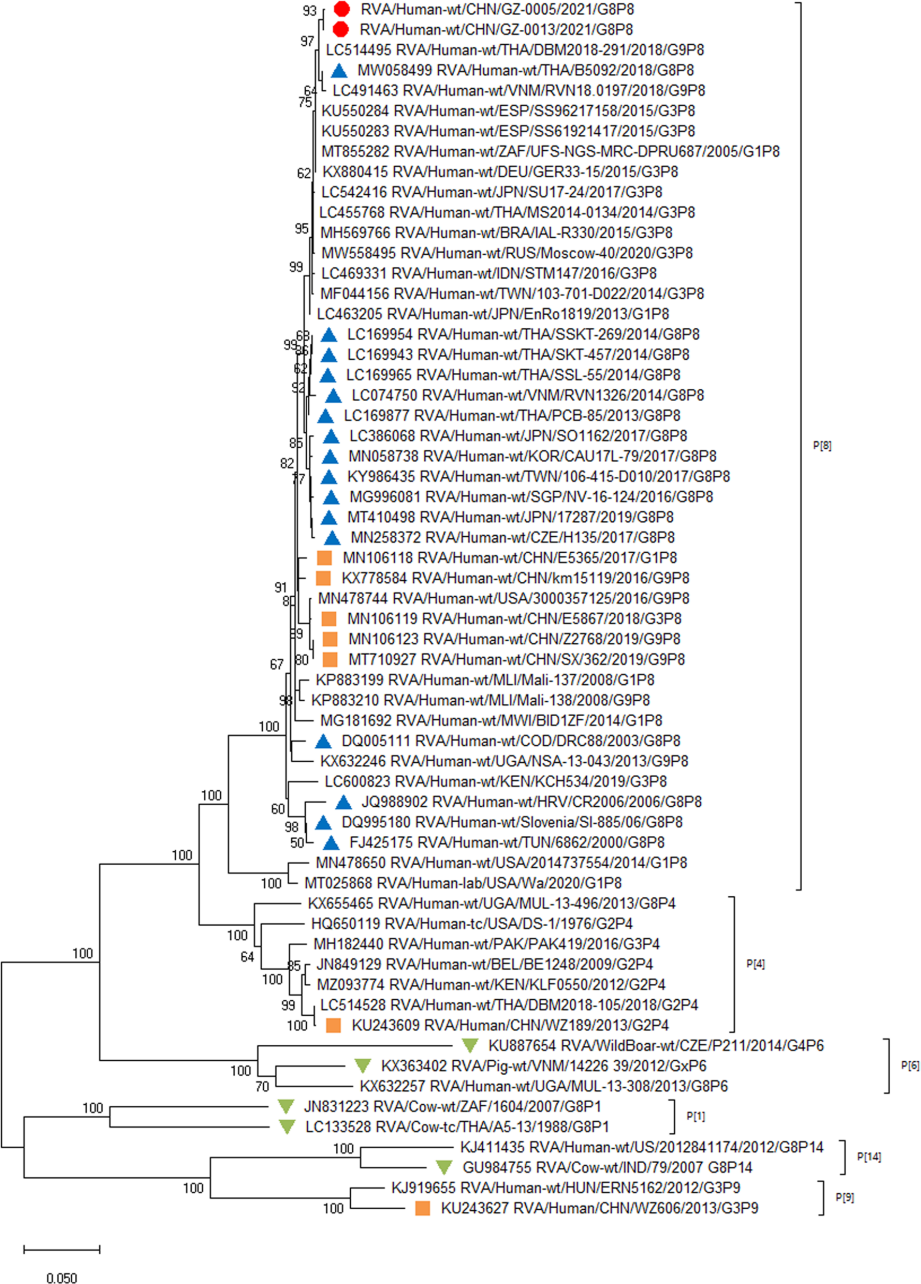
Phylogenetic analysis of the VP4 gene of G8 rotavirus strains used in the phylogenetic study of RVA strains. Phylogenetic tree of VP4 gene. This tree involves five different VP4 genes including P[8], P[4], P[6], P[14] and P[9]. Studied strains were marked in different shapes and colors.  : sample strains;  : G8P[8] strains in various regions;  : animal-derived strains;  : strains in mainland China. All sequences except those of the sample strains were obtained from the NCBI public database. The Kimura-2 parameter model was used for the construction of the Neighbor-Joining phylogenetic tree. Bootstrap numbers are shown at the branch nodes and are more reliable at values of > 70%. The scale bar indicates nucleotide substitutions per site

For VP4 genes, strain DBM2018-291/THA/G9P[8] (DS-1) circulating in Thailand had the highest sequence similarity (99.45–99.53%) (Table 2) and the closest averaged genetic distance (difference 0.0079) with the two Guangzhou strains. Analysis of G8P[8] strains circulating in Southeast Asia and East Asia from 2013 to 2019, and one strain of DS-1-like G8P[8] in the Czech Republic suggest a close genetic distance of other circulating G8P[8] strains (Fig. 2). The VP4 genes of P[8] RV strains detected in China during 2016–2019 were far less related to the two Guangzhou G8P[8] strains (Fig. 2).

### Genogrouping analysis of whole genomes and reassortment analysis

The VP1 genes of the two Guangzhou strains had the highest sequence similarity with Thailand 2018 G2P[4] strain (DS-1). The VP2, VP4, VP6, and NSP5/6 genes of the two strains had the highest sequence similarity with Thailand 2017–2018 G9P[8] strains (DS-1). The VP3, NSP1, NSP2, and NSP3 genes had the highest similarity with the Spanish G3P[8] strain in 2015 (DS-1). The most similar sequences for the two Guangzhou G8P[8] strains except for VP7 genes were found in DS-1-like G2P[4], G3P[8] and G9P[8] strains rather than other circulating G8P[8] strains. A detailed similarity score can be found in Table 2.

**Fig. 3:**
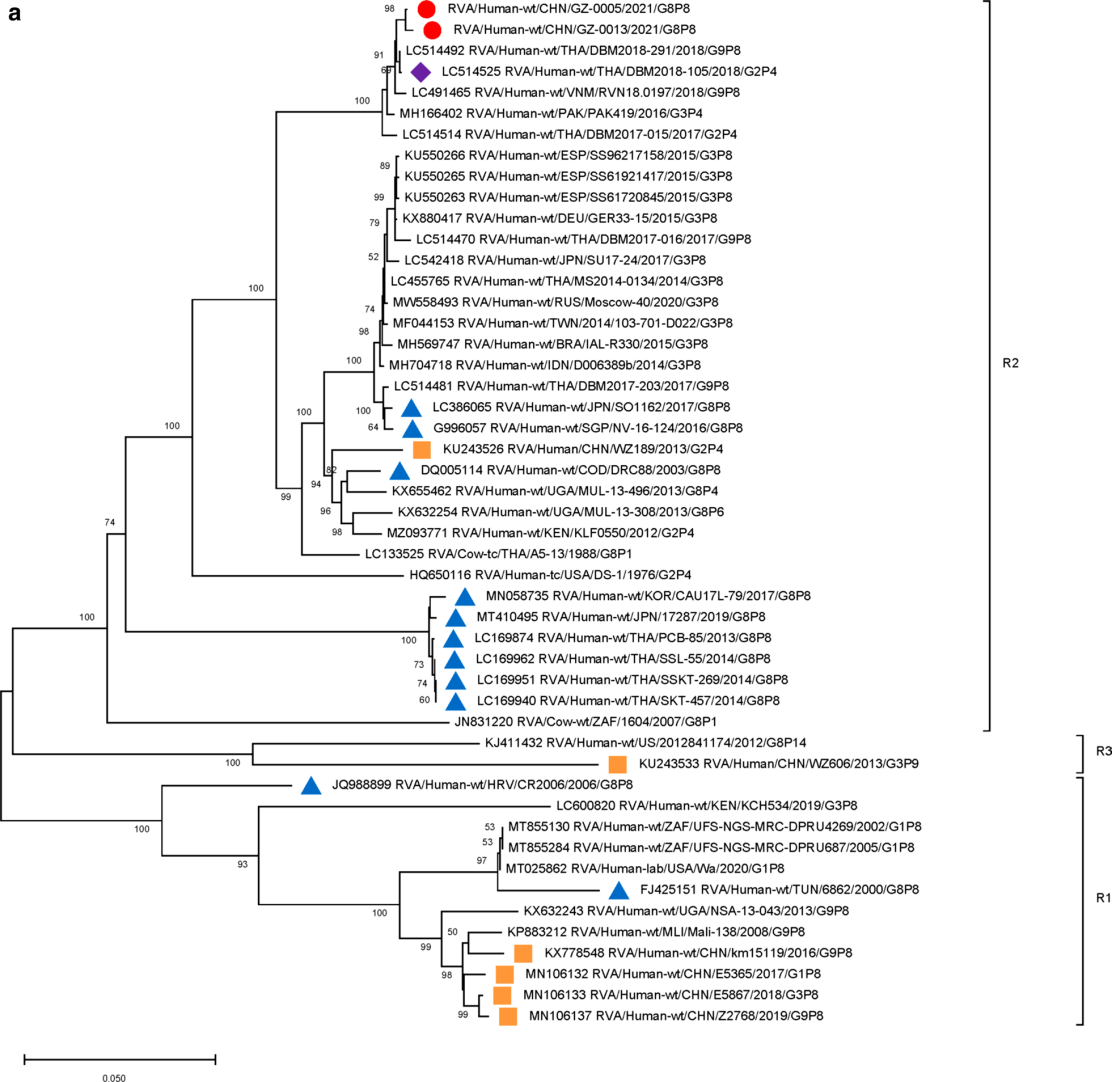

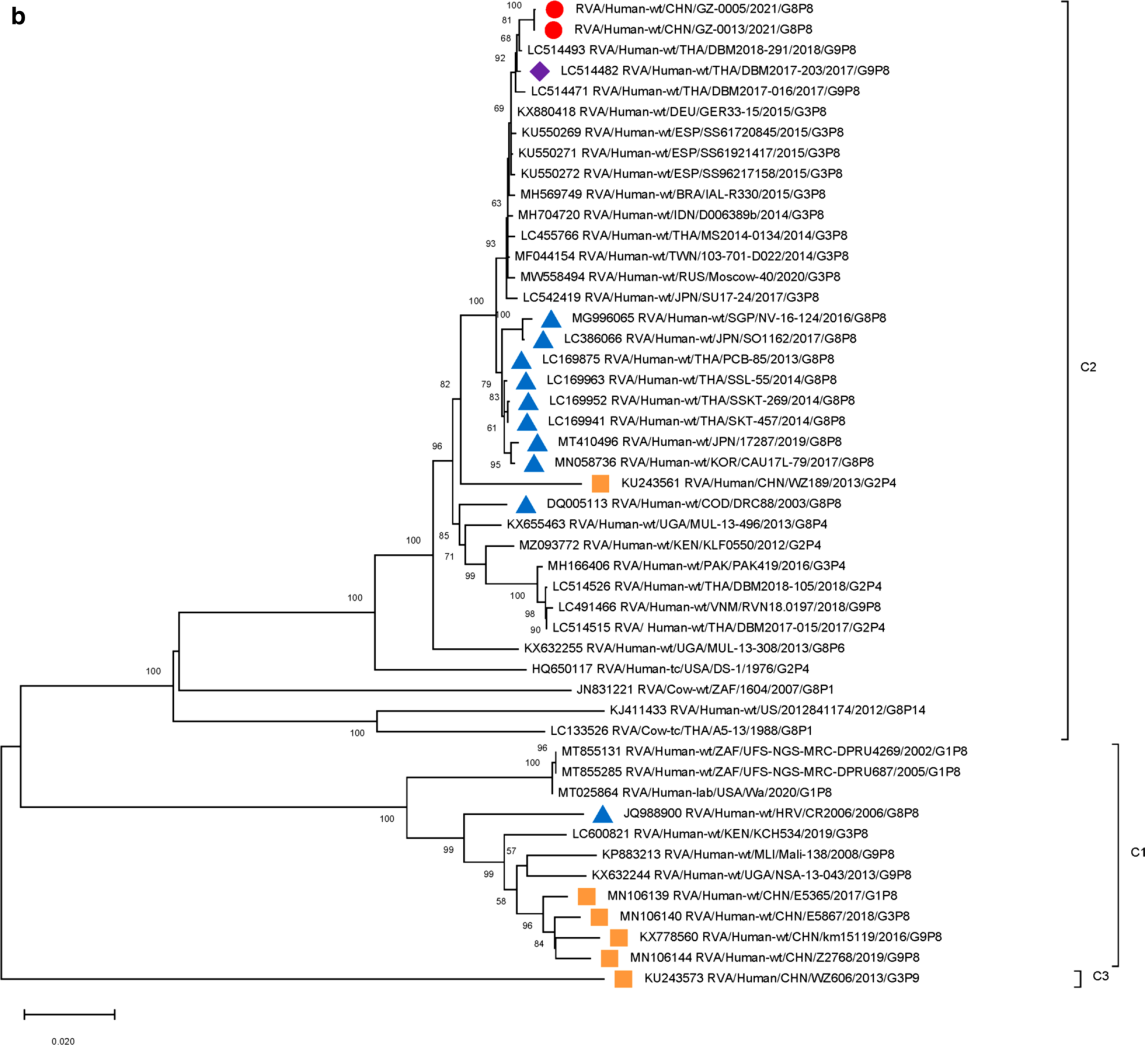

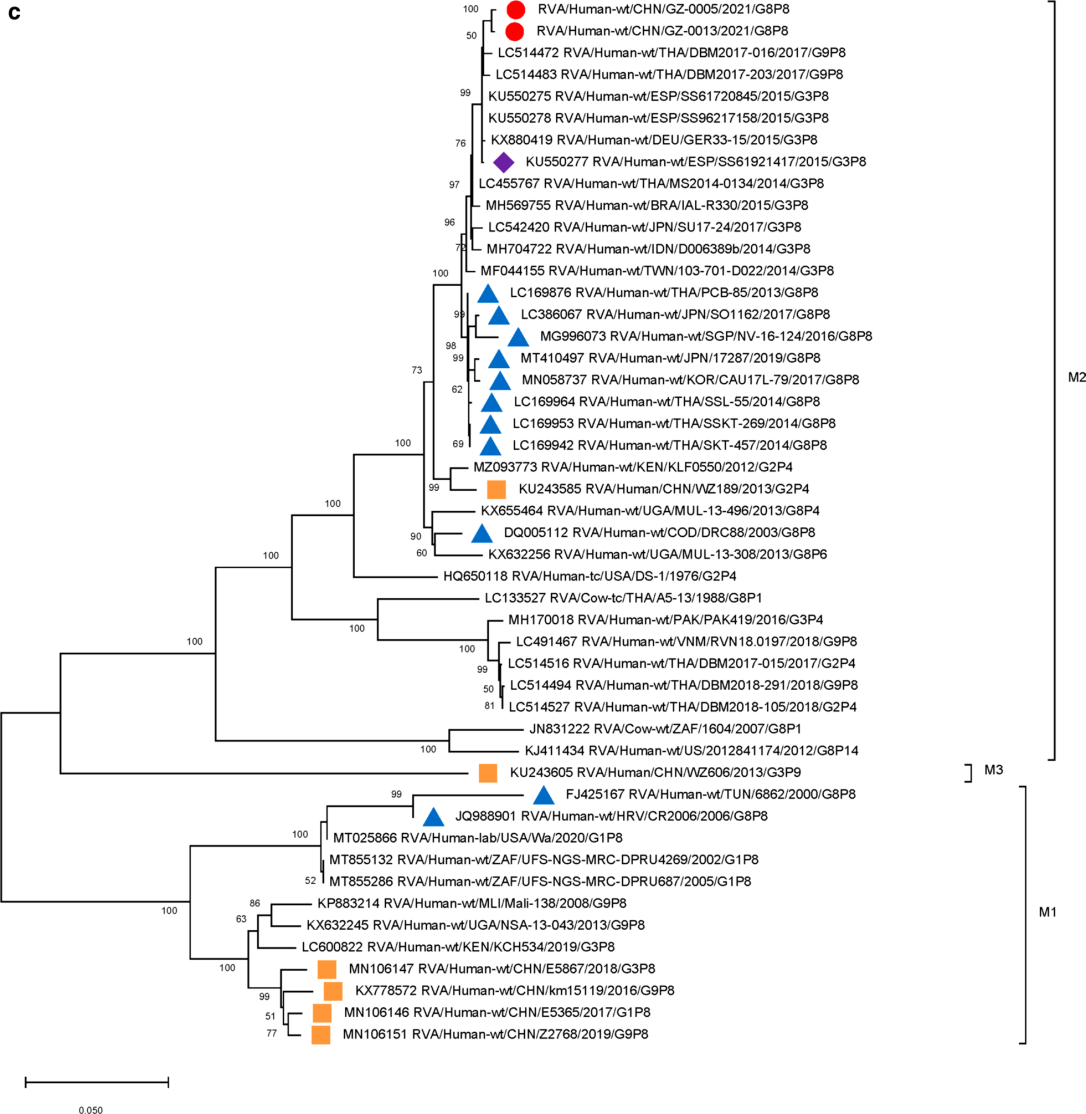

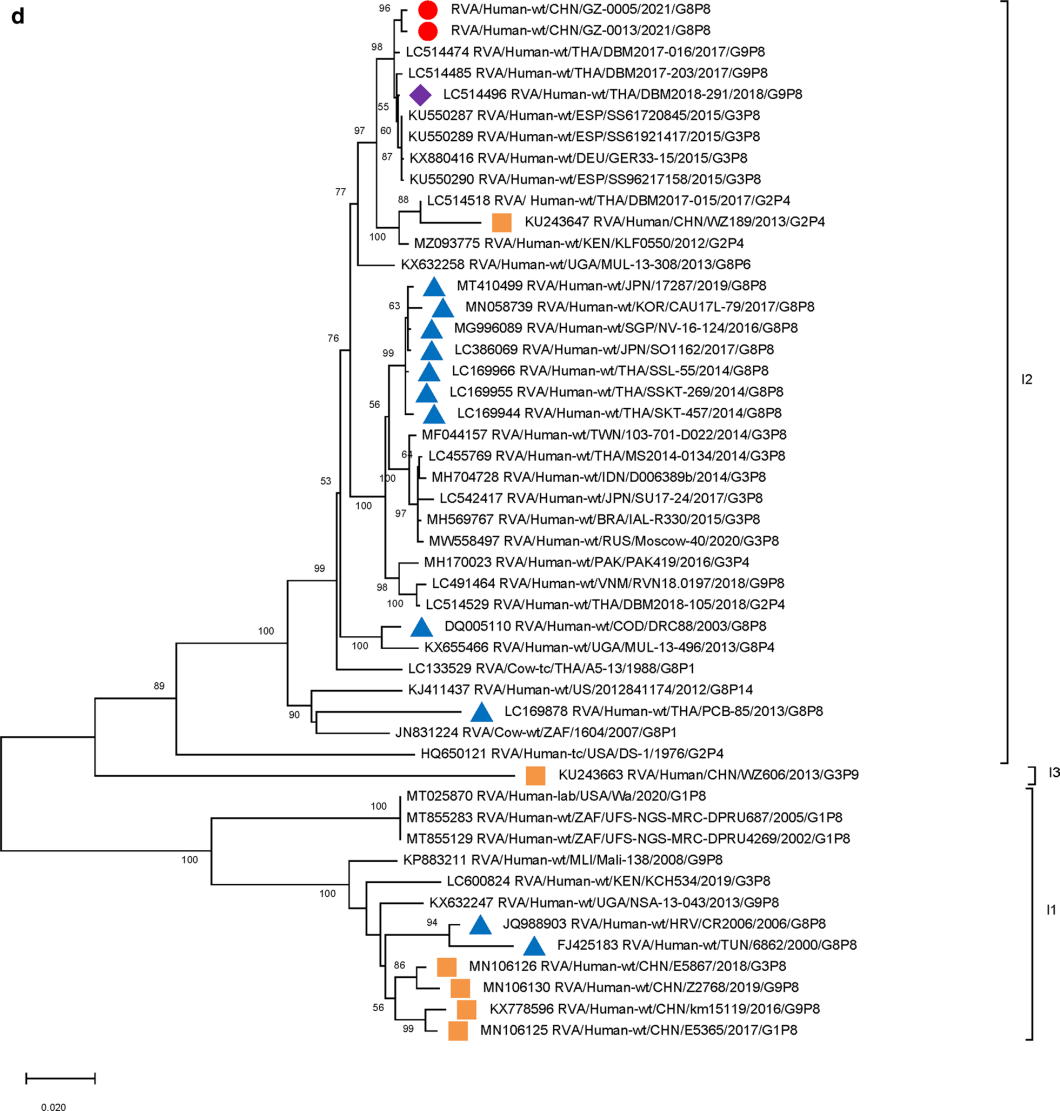

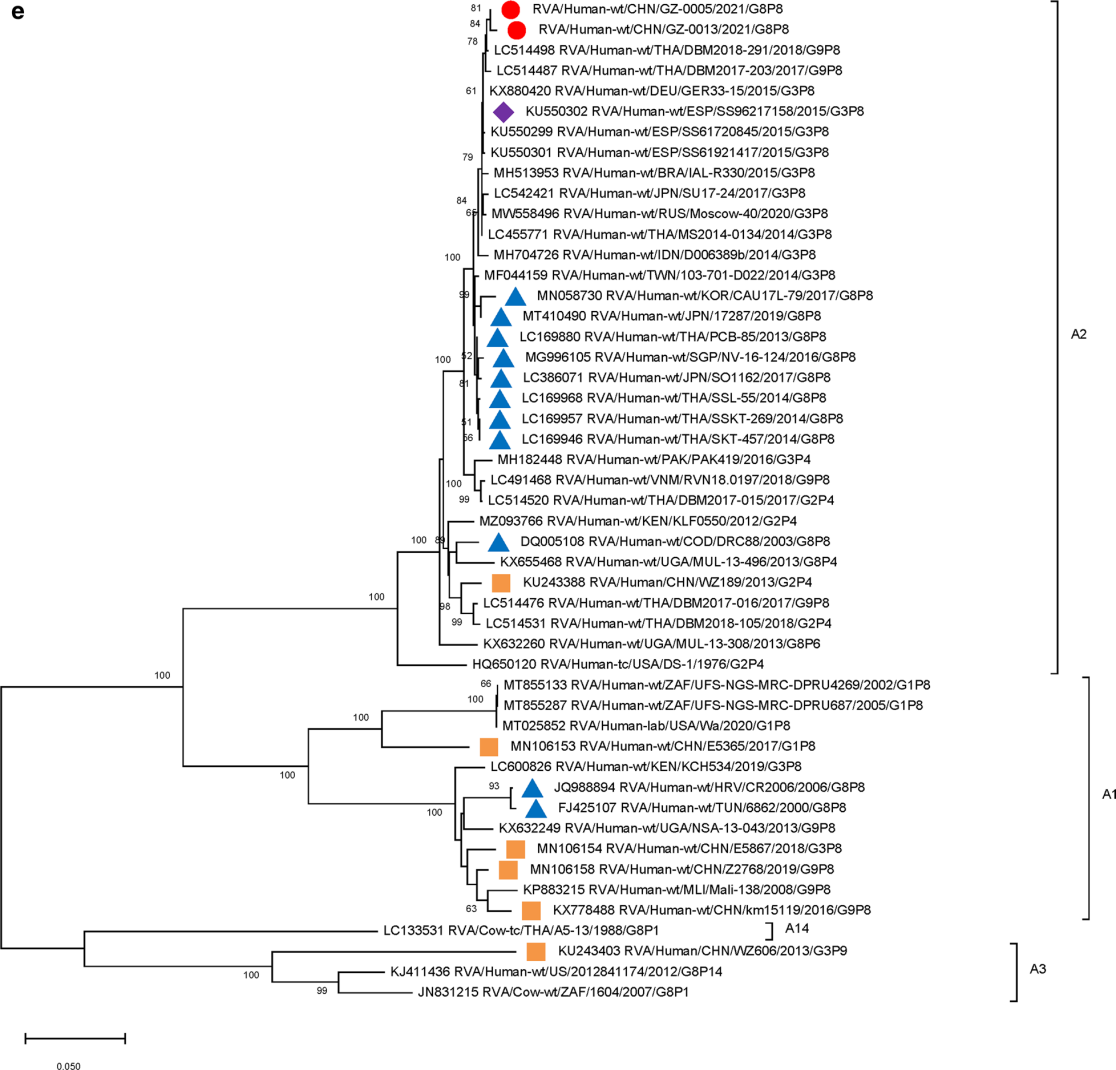

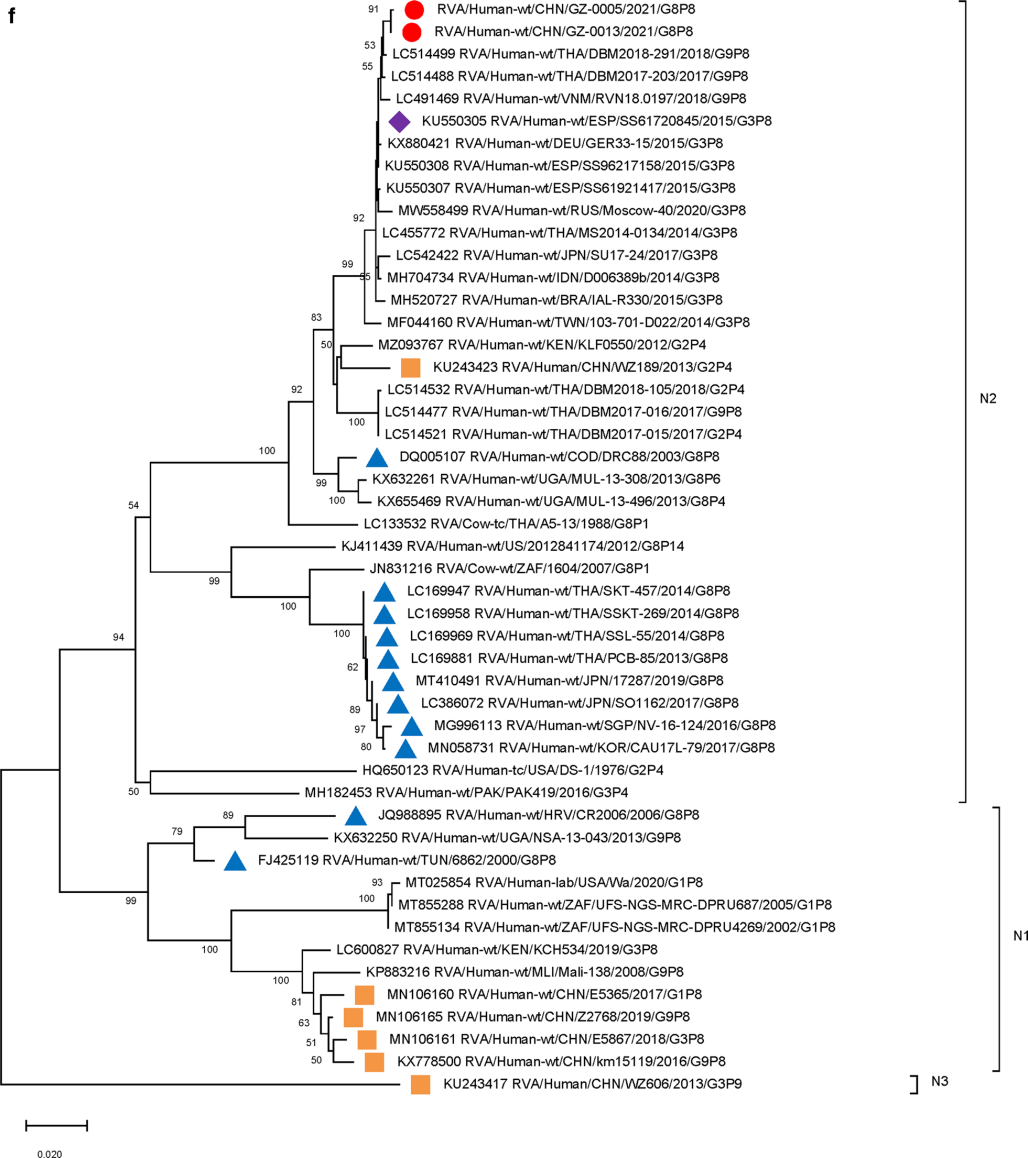

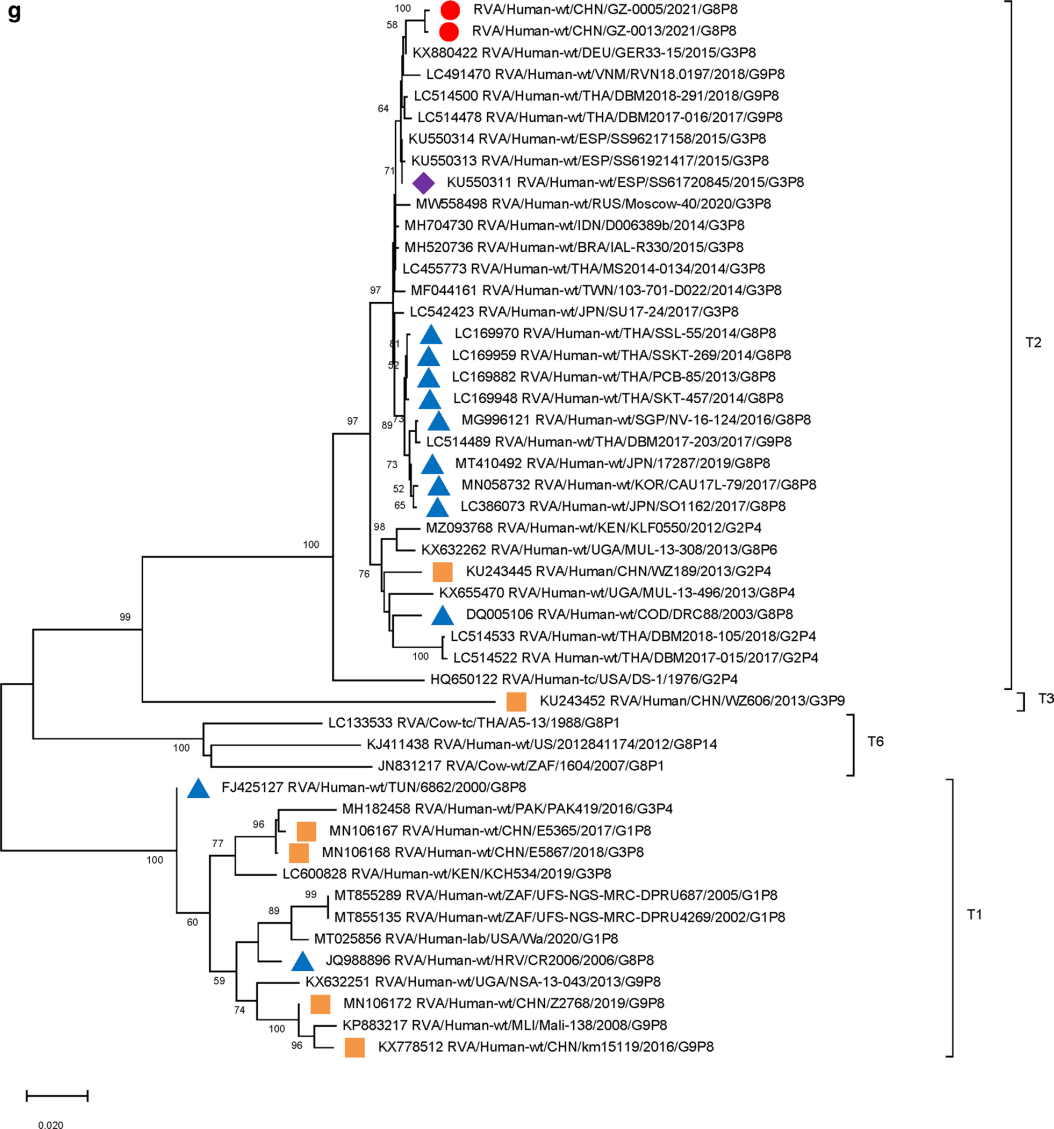

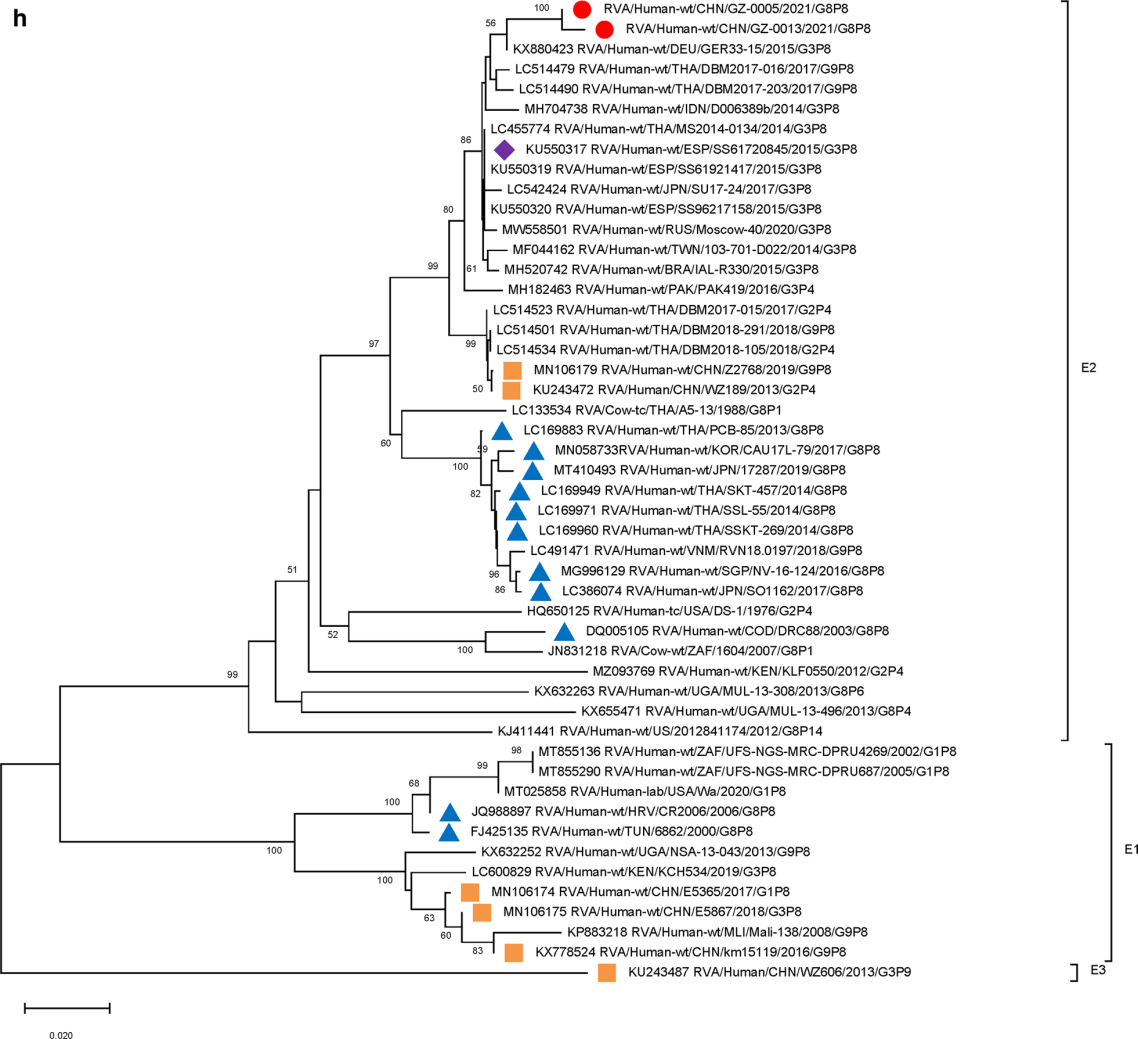

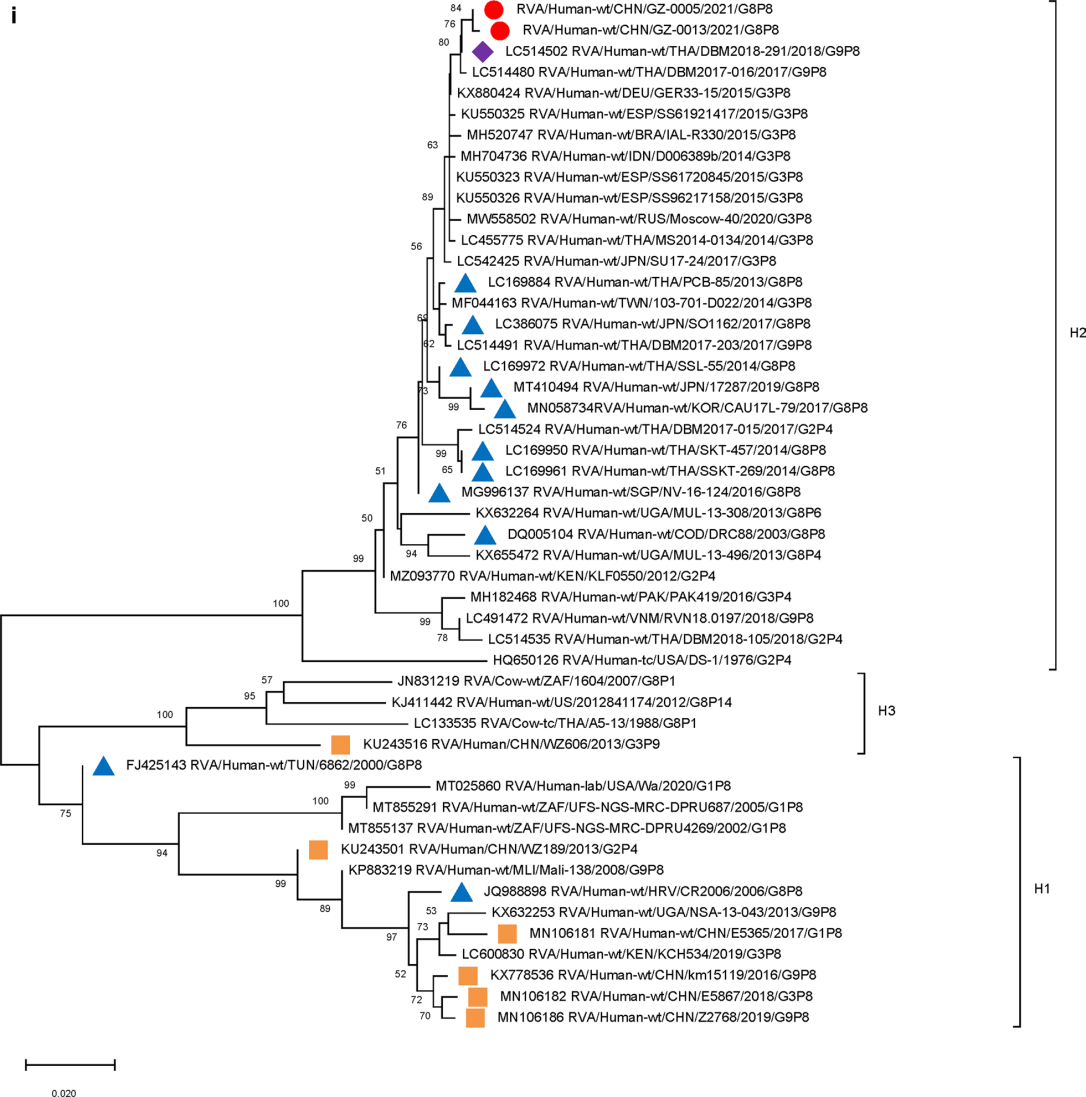
Phylogenetic trees of genome segments not encoding VP7 or VP4.**A**, VP1 gene. **B**, VP2 gene. **C**, VP3 gene. **D**, VP6 gene. **E**, NSP1 gene. **F**, NSP2 gene. **G**, NSP3 gene. **H**, NSP4 gene. **I**, NSP5/6 gene. Studied strains were marked in different shapes and colors.  : sample strains;  : G8P[8] strains in various regions;  : strains in mainland China;  : the strain with the highest similarity to the gene sequence of this segment. All sequences except those of the sample strains were obtained from the NCBI public database. The Kimura-2 parameter model was used for the construction of the Neighbor-Joining phylogenetic tree. Bootstrap numbers are shown at the branch nodes and are more reliable at values of > 70%. The scale bar indicates nucleotide substitutions per site

Furthermore, 9 genome segments other than those encoding VP7 and VP4 were analyzed through phylogenetic trees, involving the two Guangzhou G8P[8] strains and other 47 RV strains derived from GenBank (Fig. 3). For each genome segment, we focus on the location of: (1) the two Guangzhou G8P[8] strains (red dot); (2) RV strains with the highest sequence similarity with the two Guangzhou strains (purple diamond); (3) other G8P[8] strains circulating globally (blue triangle), and (4) other RV strains circulating in China (brown square). The results showed that, except for VP7 gene, the two Guangzhou G8P[8] strains did not cluster with any branch of other circulating G8P[8] strains, nor RV strains circulating in China. The VP1 (Fig. 3a), VP2 (Fig. 3b), VP4 (Fig. 2), NSP1 (Fig. 3e), NSP2 (Fig. 3f), and NSP5/6 (Fig. 3i) genome segments of the two Guangzhou strains were located in the same branch with Thailand G9P[8] strain in 2018 (DBM2018-291, DS-1). The VP3 (Fig. 3c) and VP6 (Fig. 3d) genes has the closest genetic distance to Thailand 2017 G9P[8] strains (DBM2017-016, DS-1). NSP3 (Fig. 3 g) and NSP4 (Fig. 3 h) genes were genetically the closest to GER33-15/DEU/G3P[8] (DS-1). In general, 10 genome segments except for VP7 had relatively close genetic distances with DS-1-like G9P[8], G3P[8],and G2P[4] circulating strains in Thailand, Vietnam, Spain, Germany and other places during 2015–2018 (Figs. 2 and 3).

## Discussion

This full-length genome analysis of G8P[8] RVA strains isolated in Guangzhou provided interesting results. The G8 genotype is one of the more common RV strains of bovine origin [20]. It was first discovered in humans in Indonesia between 1979 and 1981 in the form of an “ultra-short” electrophoretic pattern [21, 22]. Since then, the G8 strain has been detected in children in many countries and even became one of the dominant strains in some sub-Saharan Africa countries [23]. Even though G8 strains were circulating in multiple countries surrounding China, including India [24], Iran [25], Vietnam [26], Thailand [27], Singapore [28] and Japan [29], it was rare in China [30]. Of more than ten thousand rotavirus gene sequences submitted from China, only one strain was identified as a G8 strain (G17011060/CHN/G8P[8]) [31]. In the current study, out of five RV antigen positive samples, two were confirmed as G8P[8] strains. During the same epidemic season, a high proportion of infants with severe AGE in our ongoing multi-center RV vaccine effectiveness study were found to be infected with G8P[8] strains: Huizhou (25.0% 1/4), Shunde (55.6% 5/9), Shenzhen (42.1% 8/19) in Guangdong Province, Mianyan (36.4% 8/22) in Sichuan Province, and Xiamen (11.1% 3/27) in Fujian Province (whole-genome sequencing of these viruses had not been completed). G1, G2, G3, G4, G9 and G12 were recognized as globally important rotavirus genotypes [10, 32, 33], and studies have shown that a single novel RV (e.g., a vaccine escape mutant) can spread around the world in little more than a decade [33]. In China between 1998 and 2000, the predominant strains causing AGE in children less than five years were G1 (72.7%)[34]. After 2000, the G1 genotype decreased from 70 to 20%, while G3 type rose from 33 to 43% [35]. Around 2010, G9 strains increased and eventually replaced G3 strains [30, 36–38]. A dominant strain replacement cycle of about ten years could be inferred. These observations may indicate that the currently predominant G9 strains will be replaced by G8 strains in China. Of course, this assumption needs to be supported by further surveillance data.

Serotype G8 rotaviruses are rarely found in man and the exchange of genes between human and bovine G8 viruses may have occurred on more than one occasion [39]. G8 reassortant strains are thought to have two major lineages, one originating in Africa [40, 41], and another originating in Southeast Asia [26]. In the BEAST analysis, Hoa-Tran et al. [26] confirmed the hypothesis that the G8P[8] strains in Southeast Asia were generated by reassortment of bovine G8 strains and human DS-1-like strains and that these event occurred between 2007 and 2012. In our study, the whole genome sequencing results suggest that, although the similarity of VP7 genes between Guangzhou G8P[8] strains and bovine RVA strains derived in Southeast Asia was more than 90% (91.90 ~ 97.93%), they had higher gene homologies (99.40 ~ 99.59%) with DS-1-like G8P[8] strains circulating in Southeast Asia in recent years. It is therefore doubtful whether the two Guangzhou RV strains originated from reassortment events between animal and human strains. Secondly, the whole genome sequences of the two Guangzhou isolates differed from DS-1-like G8 and Wa-like G8 strains derived from Africa and Europe, with regard to sequence similarities and genetic distances. Thirdly, further whole genome sequence comparisons between the two Guangzhou strains and G8P[8] strains circulating in Southeast Asia and East Asia suggest a low similarity, especially regarding the VP1, VP6, NSP2 NSP4, and NSP5/6 genes. Conversely, except for the VP7 gene, higher similarity was observed with 10 other gene segments between the two Guangzhou strains and G9P[8], G3P[8] and G2P[4] strains circulating in Thailand and Spain between 2014 and 2018. Hence, it seems most likely that the two Guangzhou strains originated from reassortment events of G8P[8], G9P[8], and G3P[8] strains circulating in Southeast Asia in recent years.

One limitation of the study is that it is based on only two G8P[8] isolates obtained in Guangzhou. Further studies on prevalence, evolution and origins are required to characterize their spread in China. Nevertheless, in the past epidemic season, we have noticed the emergency of G8 strain not only in Guangzhou, but also in other regions of southern China. It would be very valuable to study the evolution-associated characteristics with more G8 strains that might spread elsewhere, to further verify our hypothesis on the origins of emerging G8P[8] RV strains in China.

## Conclusions

Probably due to the frequent personnel mobility and trade, RVAs of G8 genotype, which used to circulate in countries around China for years, have recently emerged in the South of China and accounted for a considerable proportion of children presented as severe AGE. The clinical and epidemiological significance of G8 RV strains in China remains to be closely monitored.

## Supplementary Information

**Additional file 1. Table S1.** GenBank accession numbers of Chinese rotavirus strains with G8 genotype. **Table S2.** Comparison of VP7 gene’s pairwise distances between Chinese G8 strains and closely related strains.

## Data Availability

The datasets generated during the current study are available in the GenBank sequence database. GenBank accession nos. OK349178 - OK349199.

## Abbreviations

RV: Rotavirus
AGE: Acute gastroenteritis
RT-PCR: Reverse Transcription-Polymerase Chain Reaction
