# Supplementary material for: Genetic characterization of two G8P[8] rotavirus strains isolated in Guangzhou, China, in 2020/21: evidence of genome reassortment
Source: BMC Infect Dis. 2022 Jun 28;22:579. doi: 10.1186/s12879-022-07542-9 (PMC9238253; doi:10.1186/s12879-022-07542-9)
Supplement: Supplementary file 1 — Additional file 1. Table S1. GenBank accession numbers of Chinese rotavirus strains with G8 genotype. Table S2. Comparison of VP7 gene’s pairwise distances between Chinese G8 strains and closely related strains. [file 12879_2022_7542_MOESM1_ESM.docx]

**Additional file 1: Table S1-GenBank accession numbers of Chinese rotavirus strains with G8 genotype***

| Strain | VP1 segment | VP2 segment | VP3 segment | VP4 segment | VP6 segment | VP7 segment | NSP1 segment | NSP2 segment | NSP3 segment | NSP4 segment | NSP5 segment |
| --- | --- | --- | --- | --- | --- | --- | --- | --- | --- | --- | --- |
| GZ-0005 | OK349178 | OK349179 | OK349180 | OK349181 | OK349182 | OK349183 | OK349184 | OK349185 | OK349186 | OK349187 | OK349188 |
| GZ-0013 | OK349189 | OK349190 | OK349191 | OK349192 | OK349193 | OK349194 | OK349195 | OK349196 | OK349197 | OK349198 | OK349199 |

**Additional file 1: Table S2-Comparison of VP7 gene’s pairwise distances between Chinese G8 strains and closely related strains**

| Strain | CHN/GZ-0005/2021/G8P[8] | CHN/GZ-0013/2021/G8P[8] |
| --- | --- | --- |
| DS-1-like G8 (Southeast Asia) |  |  |
| RVA/Human-wt/THA/SSL-55/2014/G8P8 | 0.0066 | 0.0086 |
| RVA/Human-wt/JPN/17287/2019/G8P8 | 0.0067 | 0.0086 |
| RVA/Human-wt/THA/SKT-457/2014/G8P8 | 0.0076 | 0.0095 |
| RVA/Human-wt/JPN/SO1162/2017/G8P8 | 0.0081 | 0.0102 |
| RVA/Human-wt/SGP/NV-16-124/2016/G8P8 | 0.0083 | 0.0104 |
| RVA/Human-wt/KOR/CAU17L-79/2017/G8P8 | 0.0085 | 0.0105 |
| RVA/Human-wt/THA/PCB-85/2013/G8P8 | 0.0095 | 0.0114 |
| DS-1-like G8 (African & Europe) |  |  |
| RVA/Human-wt/COD/DRC88/2003/G8P8 | 0.1476 | 0.1476 |
| RVA/Human-wt/UGA/MUL-13-308/2013/G8P6 | 0.1488 | 0.1488 |
| RVA/Human-wt/UGA/MUL-13-496/2013/G8P4 | 0.1500 | 0.1500 |
| RVA/Human-wt/MWI/BID1B9/2012/G8P4 | 0.1560 | 0.1560 |
| RVA/Human-wt/KEN/KDH1255/2012/G8P4 | 0.1567 | 0.1567 |
| RVA/Human-wt/COD/DRC86/2003/G8P6 | 0.1678 | 0.1678 |
| Wa- like G8 |  |  |
| RVA/Human-wt/HRV/CR2006/2006/G8P8 | 0.1740 | 0.1689 |
| RVA/Human-wt/USA/2009727045/2009/G8P4 | 0.1887 | 0.1834 |
| RVA/Human-wt/SVN/SI-885/06/G8P8 | 0.1965 | 0.1899 |
| RVA/Human-wt/TUN/6862/2000/G8P8 | 0.1966 | 0.1901 |
